# Demethylation initiated by ROS1 glycosylase involves random sliding along DNA

**DOI:** 10.1093/nar/gks894

**Published:** 2012-10-02

**Authors:** María Isabel Ponferrada-Marín, Teresa Roldán-Arjona, Rafael R. Ariza

**Affiliations:** Department of Genetics, University of Córdoba/IMIBIC, 14071 Córdoba, Spain

## Abstract

Active DNA demethylation processes play a critical role in shaping methylation patterns, yet our understanding of the mechanisms involved is still fragmented and incomplete. REPRESSOR OF SILENCING 1 (ROS1) is a prototype member of a family of plant 5-methylcytosine DNA glycosylases that initiate active DNA demethylation through a base excision repair pathway. As ROS1 binds DNA non-specifically, we have critically tested the hypothesis that facilitated diffusion along DNA may contribute to target location by the enzyme. We have found that dissociation of ROS1 from DNA is severely restricted when access to both ends is obstructed by tetraloops obstacles. Unblocking any end facilitates protein dissociation, suggesting that random surface sliding is the main route to a specific target site. We also found that removal of the basic N-terminal domain of ROS1 significantly impairs the sliding capacity of the protein. Finally, we show that sliding increases the catalytic efficiency of ROS1 on 5-meC:G pairs, but not on T:G mispairs, thus suggesting that the enzyme achieves recognition and excision of its two substrate bases by different means. A model is proposed to explain how ROS1 finds its potential targets on DNA.

## INTRODUCTION

DNA methylation at carbon 5 of cytosine 5-methylcytosine (5-meC) is a reversible epigenetic mark that influences chromatin structure and is usually associated with gene silencing (1), although its effects may vary in different genomic contexts (2). In eukaryotes, DNA methylation is involved in vital physiological processes such as genomic imprinting, X chromosome inactivation, defence against parasitic mobile elements and the establishment of developmental programs (3). In agreement with such essential roles, aberrant DNA methylation has important consequences for cells and is a crucial component in many forms of human disease, including cancer (4,5).

Methylation patterns are the dynamic outcome of antagonistic methylation and demethylation processes, but until recently only the former were known to some detail (6). In plants, active DNA demethylation is initiated by a group of DNA glycosylases, typified by *Arabidopsis* REPRESSOR OF SILENCING (ROS1) and DEMETER (DME) (7,8). ROS1 and DME remove 5-meC as a free base and recruit the base excision repair machinery to fill in the gap with an unmethylated cytosine (9–11). Animals apparently lack 5-meC DNA glycosylases, but several lines of evidence suggest that demethylation involves excision of de-aminated and/or oxidized derivatives of 5-meC [reviewed in (12)].

Plant 5-meC DNA glycosylases are bifunctional enzymes with an associate lyase activity that cleaves the phosphodiester backbone at the 5-meC removal site by β,δ-elimination, generating as a major product a single-nucleotide gap flanked by 3′-phosphate and 5′-phosphate termini (9,11,13). This gap is further processed by the DNA 3′-phosphatase ZDP, which removes the blocking 3′-phosphate and allows subsequent DNA polymerization and ligation steps by additional proteins of the base excision repair pathway (14). *In vivo*, DME demethylates the maternal allele of imprinted genes in the endosperm (10), whereas ROS1 and its paralogs DML2 and DML3 counteract excessive methylation at several hundred loci across the genome (13,15,16). In addition to 5-meC, the proteins of the ROS1/DME family also remove T mismatched to guanine (10,11,13), thus suggesting an additional role in neutralizing the mutagenic consequences of the spontaneous deamination of 5-meC (17).

ROS1 and its homologs belong to the HhH-GPD (helix-hairpin-helix followed by a Gly-Pro rich loop and a conserved Asp) superfamily, the largest and most functionally diverse group of DNA glycosylases (18). This superfamily is widespread in bacteria, archaea and eukaryotes, and its members are typically 200–400 amino acids long (19). However, ROS1/DME proteins appear to be plant specific, are unusually large (1100–2000 amino acids) and, unlike other DNA glycosylases, possess a bipartite catalytic domain divided by a large low-complexity insert predicted to have an unstructured conformation (20). In addition to their discontinuous DNA glycosylase domain, members of the ROS1/DME family share a carboxy-terminal domain of unknown function (11) and a short N-terminal domain significantly rich in lysine. This basic domain mediates strong methylation-independent binding of ROS1 to DNA and greatly facilitates 5-meC excision on long DNA substrates (21). Based on these results, we have suggested that 5-meC recognition involves initial non-specific binding events to non-target sites followed by facilitated diffusion along DNA (21).

The reduction in dimensionality afforded by facilitated diffusion was originally proposed as a way to speed up target site location in the *Escherichia coli* lac repressor-operator system (22). This process may combine a one-dimensional (1D) diffusion of the protein along the contour length of DNA, called sliding, and short- or long-range three-dimensional (3D) intramolecular excursions named hopping or jumping, respectively (23). Facilitated diffusion has found experimental support in a number of proteins, including restriction endonucleases (24), DNA methyltransferases (25) and DNA glycosylases (26,27). Direct evidence of fast diffusion along DNA by several DNA glycosylases has been obtained in single-molecule studies (28,29). However, the resolution limits of such methods do not allow direct discrimination between 1D sliding along the contour length of the DNA and intramolecular hopping.

In this study, we analysed ROS1 binding capacity and catalytic activity on DNA substrates containing tetraloops blocks that impede protein sliding but should not affect alternative modes of target search, such as 3D diffusion or intramolecular hopping/jumping. Our results indicate that ROS1 performs facilitated diffusion through random 1D sliding along DNA, and that this process requires the basic N-terminal domain of the protein. Furthermore, we found that sliding speeds up processing of 5-meC:G pairs, but not T:G mispairs, thus suggesting that ROS1 achieves recognition and excision of its two target bases by different means.

## MATERIALS AND METHODS

### DNA substrates

**Figure 1.**
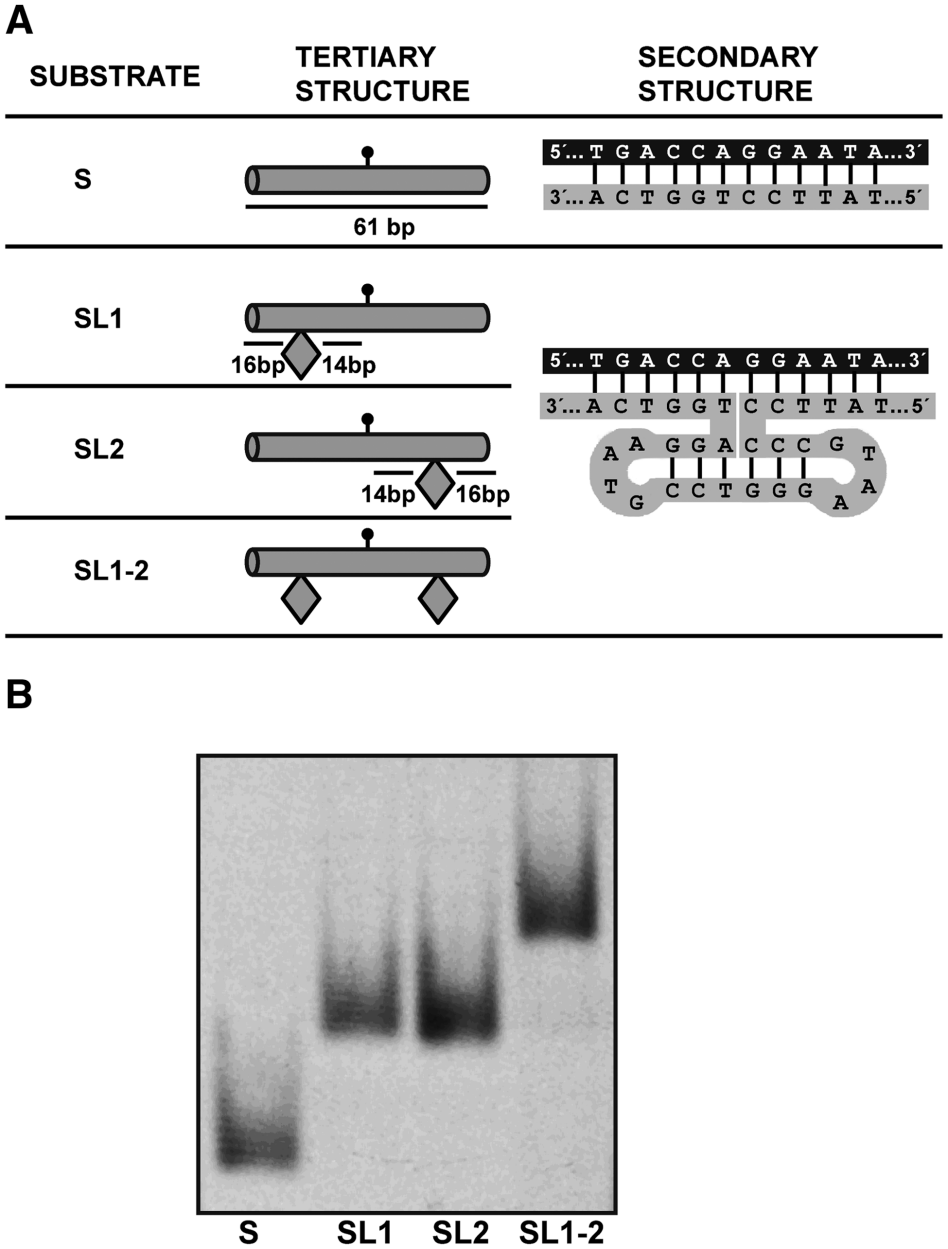
Overview of DNA substrates. (**A**) DNA substrate design. Tertiary structure is shown schematically, indicating obstacle positions relative to the target residue location (marked by a lollipop). Secondary structure diagrams show the intended base pairing of the DNA substrates. Obstacles in substrates SL1, SL2 and SL1-2 are created by a 6-bp DNA helix capped at both ends with tetraloops, which is connected to the DNA substrate via a four-way junction (see text for details and Supplementary Table S1 for oligonucleotide sequences). Schematic diagrams are adapted from (30). (**B**) Relative mobilities of DNA substrates. Fluorescein-labe**l**led substrates S, SL1, SL2 and SL1-2 were subjected to electrophoresis at 100 V for 4 h in a 5% non-denaturing polyacrylamide gel.

Oligonucleotides used as DNA substrates (Table S1) were synthesized by Operon and purified by PAGE before use. Double-stranded DNA substrates were prepared by mixing a 5-μM solution of a 5′-fluorescein-labelled oligonucleotide (upper strand) with a 10-μM solution of an unlabelled oligomer (lower strand), heating to 95°C for 5 min and slowly cooling to room temperature. In substrates SL1, SL2 and SL1-2, an obstacle is created by a 6-bp DNA helix capped at both ends with tetraloops, which is connected to the DNA substrate via a four-way junction (Figure 1). These substrates were obtained by annealing two oligonucleotides of different lengths. The strands were complementary at their ends, but the longer strand contained an additional interior stretch of 20 nucleotides that was designed to fold into the obstacle described earlier.

### Expression and purification of recombinant proteins

Full-length ROS1 (FL-ROS1) and its N-terminal truncated version were expressed and purified as N-terminal His-tagged proteins, as previously described (21,31).

### Enzyme activity assays

Fluorescein-labelled duplex oligonucleotides (20 nM) were incubated at 30°C for the indicated times in a reaction mixture containing 50 mM Tris–HCl pH 8.0, 1 mM EDTA, 1 mM DTT, 0.1 mg/mL BSA, and the indicated amounts of FL-ROS1 or the mutant variant in a total volume of 50 μL. Reactions were stopped by adding 20 mM EDTA, 0.6% sodium dodecyl sulphate and 0.5 mg/mL proteinase K, and the mixtures were incubated at 37°C for 30 min. DNA was extracted with phenol:chloroform:isoamyl alcohol (25:24:1), and ethanol precipitated at −20°C in the presence of 0.3 mM NaCl and 16 μg/mL glycogen. Samples were re-suspended in 10 μL 90% formamide and heated at 95°C for 5 min. Reaction products were separated in a 12% denaturing polyacrylamide gel containing 7 M urea. Fluorescein-labelled DNA was visualized in a FLA-5100 imager and analysed using Multigauge software (Fujifilm).

FL-ROS1 does not exhibit significant turnover *in vitro* owing to strong product binding, and therefore a simple Michaelis–Menten model is inadequate for a correct kinetic analysis of this enzyme. As we have previously described (20,21,31), the standard reaction conditions were equimolar (20 nM) enzyme/substrate ratios and incubation at 30°C. Data were fitted to the equation [Product] = P_max_ [1-exp^(-kt)^] using non-linear regression analysis and the software Sigmaplot, and the parameters P_max_ (maximum substrate processing within an unlimited period of time), T_50_ (the time required to reach 50% of the product plateau level, P_max_) and relative processing efficiency (E_rel_ = P_max_/T_50_) were determined (32). A representative example of 5-meC DNA glycosylase assay and kinetic analysis is shown in Supplementary Figure S1.

### Electrophoretic mobility shift assay

Band-shift reactions were performed using fluorescein-labelled duplex oligonucleotides. In standard gel-retardation reactions, increasing amounts of FL-ROS1 were incubated with 100 nM fluorescein-labelled oligonucleotide substrates. Competition bandshift reactions were performed by pre-incubating FL-ROS1 (120 nM) and NΔ294-ROS1 (200 nM) with 100 nM fluorescein-labelled substrates at 25°C for 5 min and then adding increasing amounts of unlabelled duplex S as competitor. DNA binding reactions were carried out at 25°C for 60 min in 10 nM Tris–HCl pH 8.0, 1 mM DTT, 10 μg/ml BSA, 1 mM EDTA in a final volume of 10 μl. Complexes were electrophoresed through 0.2% agarose gels in 1 × TAE (40 mM Tris-acetate, 1 mM EDTA, pH 8.0). Electrophoresis was carried out in 1 × TAE for 40 min at 80 V at room temperature. Fluorescein-labelled DNA was visualized in a FLA-5100 imager and analysed using Multigauge software (Fujifilm).

## RESULTS

### DNA substrates design

To test whether ROS1 is capable of linear diffusion along DNA, we constructed four substrates (Figure 1) whose design was inspired on a previously published study on obstacle bypass by EcoRI (30). All four substrates contain a 5-meC:G pair (or a T:G mispair, when indicated) at a central position in a 5′-CG-3′ context (Figure 1A). Substrate S is a control 61-mer DNA duplex with no obstacles. Substrate SL1-2 contains two identical obstacles, each 14 pb away from the target residue. Substrates SL1 and SL2 contain only one obstacle either on the left or the right side, respectively. Both the space between obstacles (29 pb) and their distance to the nearest DNA terminus (16 bp) (Figure 1A) are above the minimal DNA substrate length (14 bp) required for ROS1 catalytic activity (21).

Substrates SL1, SL2 and SL1-2 were obtained by annealing two oligonucleotides of different lengths (Supplementary Table S1). The obstacles consist of a 20-nt loop created by a 6-bp DNA helix capped at both ends with GTAA tetraloops, which are connected to the DNA substrate via a four-way junction (30) (Figure 1A). This structure is known to be particularly stable owing to the presence of 5 C:G pairs at the loop-closing positions (33). All four substrates were analysed by non-denaturing polyacrylamide gel electrophoresis (Figure 1B). We found that substrate SL1-2 migrates more slowly than substrates SL1 and SL2, which in turn show lower mobility than substrate S. Therefore, the relative mobility of the four DNA substrates during native gel electrophoresis agrees with their predicted secondary structures. Although it cannot be excluded that the short 20-pb helix forming the obstacle assumes a slightly different structure, it will represent in any case an obstruction to 1D sliding along DNA.

### ROS1 slides along DNA

**Figure 2.**
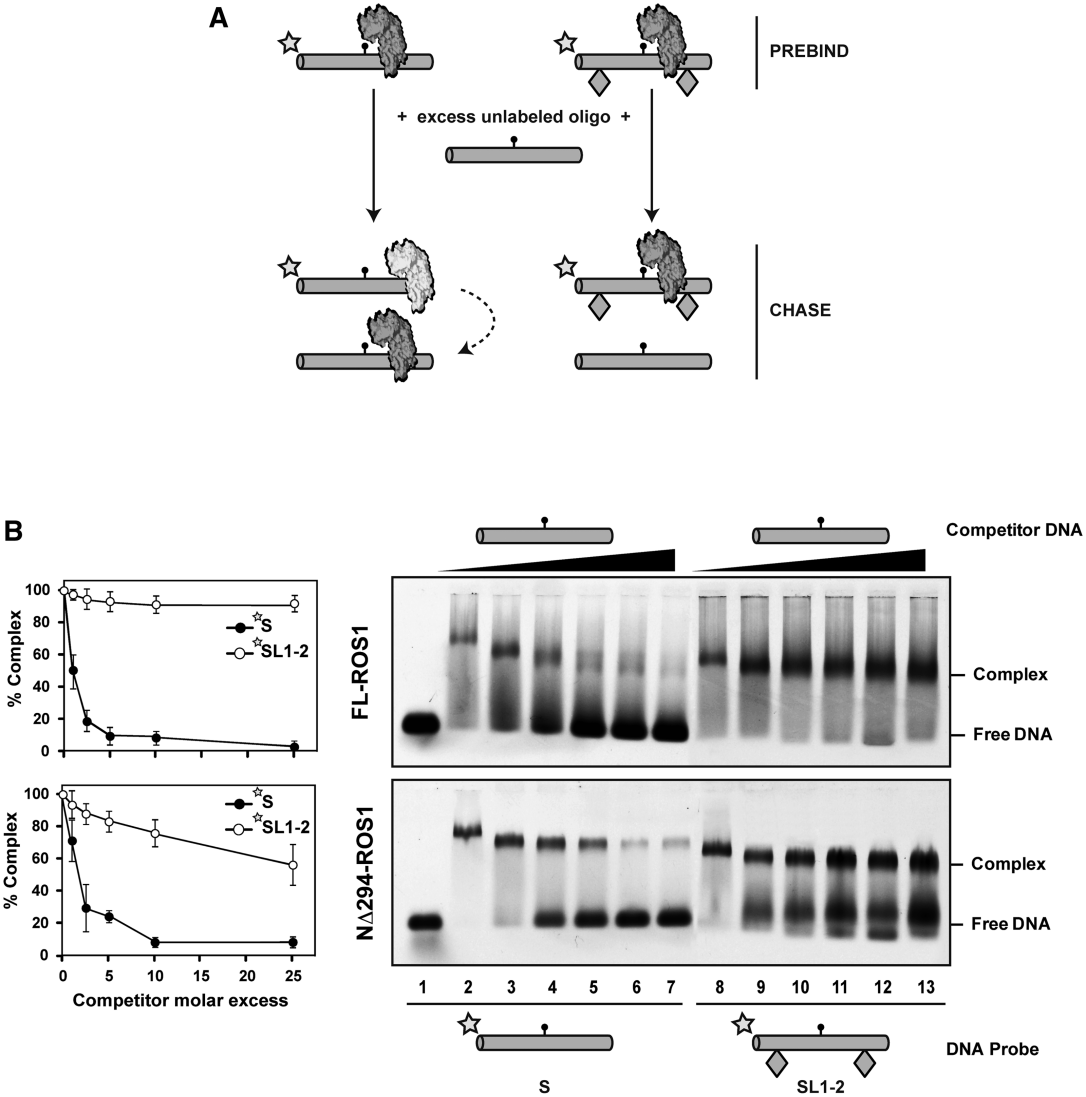
ROS1 slides DNA in a process that requires its N-terminal domain. (**A**) Schematic diagram showing the experimental setup used to assay for linear diffusion. Proteins were pre-incubated for 5 min with fluorescein-labelled substrates S or SL1-2, both containing a single 5-meC:G pair, and then chased by addition of unlabelled S competitor. If linear diffusion occurs, dissociation will be faster from labelled substrate S. (**B**) Gel shift assay showing dissociation of FL-ROS1 (upper panel, 120 nM) and NΔ294-ROS1 (lower panel, 200 nM) from fluorescein-labelled substrates S (lanes 2–7, 100 nM) and SL1-2 (lanes 8–13, 100 nM) on addition of increasing amounts (0, 0.1, 0.25, 0.5, 1.0 and 2.5 μM) of methylated unlabelled competitor S. After non-denaturing gel electrophoresis, the gel was scanned to detect fluorescein-labelled DNA. Protein–DNA complexes were identified by their retarded mobility compared with that of free DNA, as indicated. Graphs on the left show the percentage of remaining complex versus competitor molar excess ratios. Values are mean ± SE from two independent experiments, adjusted with the unbiased estimator described in (36).

To test whether ROS1 performs linear diffusion while searching for its target base, we adapted a previously described electrophoretic mobility shift assay (EMSA) competition assay (34,35). We pre-incubated FL-ROS1 with fluorescein-labelled substrates S or SL1-2 and then added increasing concentrations of unlabelled S competitor to promote dissociation. If, once bound to DNA, ROS1 searches its target strictly by linear diffusion, the protein would slide off the DNA with free ends (labelled S probe), but not the DNA with ends occluded by obstacles (labelled SL1-2 probe). However, if the protein uses a 3D search mechanism, it would dissociate frequently from the central portion of both S and SL1-2 probes, and therefore the presence of obstacles would just slightly affect its dissociation rate (Figure 2A).

We found that FL-ROS1 dissociates from substrate S when chased by the competitor, but remains bound to substrate SL1-2 even at high competitor concentrations (Figure 2B, upper panel). We confirmed that the inability of ROS1 to dissociate from SL1-2 probe is not due to a higher affinity for this substrate compared with substrate S (Supplementary Figure S2). Similar results to those shown in Figure 2B were obtained when the substrates S and SL1-2 lacked a target residue (Supplementary Figure S3). In agreement with this, we found that binding and dissociation of ROS1 are independent of catalytic activity (Supplementary Figure S4). A higher dissociation rate both from the S and SL1-2 labelled substrates was observed when using SL1-2 as competitor, compared with S (Supplementary Figure S5), probably because an excess of SL1-2 competitor reduces the chance of re-binding the probe. Altogether, these results indicate that ROS1 dissociation occurs primarily, although not exclusively, from DNA ends, and strongly suggest that the protein performs sliding on DNA while searching for its target base.

### DNA sliding is facilitated by the N-terminal domain of ROS1

We next examined whether there is a protein region necessary for DNA sliding. We have previously reported that deletion of the N-terminal, lysine-rich domain of ROS1 produces a protein (NΔ294-ROS1) with a reduced DNA-binding capacity compared with the full-length version (21). Interestingly, such N-terminal truncated protein demethylates short substrates (20 pb) with an efficiency similar to FL-ROS1, but shows a significantly lower activity on long DNA molecules (52 pb) (21). Based on these observations, we have previously proposed that the non-specific DNA binding afforded by the basic N-terminus may assist the enzyme to scan the DNA in 1D diffusion to locate its target base (21).

To test this hypothesis, we used NΔ294-ROS1 in an EMSA competition assay analogous to that described earlier (Figure 2B, lower panel). We found that, as previously observed with FL-ROS1, the truncated version easily dissociates from substrate S when chased, although with slightly lower dissociation rates at low concentrations of competitor. However, in stark contrast to the full-length version, NΔ294-ROS1 does not remain bound to the SL1-2 substrate when chased, and it is significantly displaced from DNA at high competitor concentrations (Figure 2B, lower panel). We performed titration experiments that showed that NΔ294-ROS1 binds at lower concentrations to SL1-2 compared with S (Supplementary Figure S6). Interestingly however, although NΔ294-ROS1 binds at lower concentrations than FL-ROS1 to SL1-2 (compare Supplementary Figures S2 and S6), once bound to the substrate with obstacles NΔ294-ROS1 is easier to dissociate than FL-ROS1 by competition. Altogether, these results suggest that the positively charged N-terminal domain plays a role in stabilizing the protein-DNA complex and facilitates sliding.

### ROS1 slides DNA randomly

**Figure 3.**
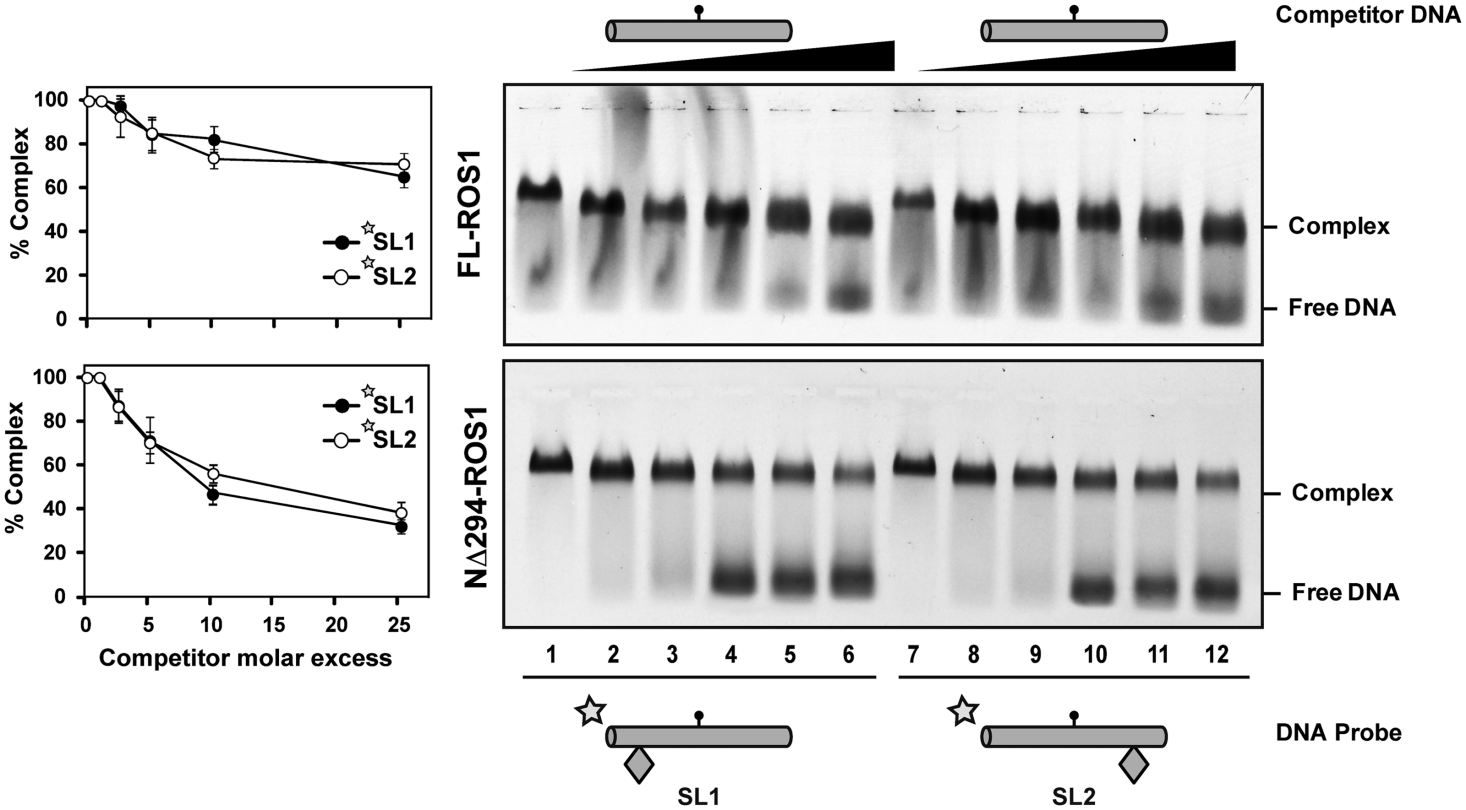
ROS1 slides DNA randomly. Gel shift assay showing dissociation of FL-ROS1 (upper panel, 120 nM) and NΔ294-ROS1 (lower panel, 200 nM) from fluorescein-labelled substrates SL1 (lanes 2–7, 100 nM) and SL2 (lanes 8–13, 100 nM) containing a single 5-meC:G pair on addition of increasing amounts (0, 0.1, 0.25, 0.5, 1.0 and 2.5 μM) of methylated unlabelled competitor S. After non-denaturing gel electrophoresis, the gel was scanned to detect fluorescein-labelled DNA. Protein-DNA complexes were identified by their retarded mobility compared with that of free DNA, as indicated. Graphs on the left show the percentage of remaining complex versus competitor molar excess ratios. Values are mean ± SE from two independent experiments, adjusted with the unbiased estimator described in (36).

We next tested whether ROS1 exhibits any sort of directionality during DNA sliding. To this end, we performed EMSA competition assays with substrates SL1 and SL2, each containing a single obstacle on either side of the target 5-meC residue (Figure 3). We found that FL-ROS1 exhibits equally low dissociation rates on both substrates, although such rates were slightly higher than that observed with the SL1-2 substrate (compare upper panels from Figures 3 and 2B). We also followed the dissociation of ROS1 from SL1 or SL2 at different time points in competition with unlabelled SL1-2 and did not find significant differences between both probes (Supplementary Figure S7). These observations suggest that un-blocking one end, regardless of its position, facilitates protein dissociation from DNA. We also tested the behaviour of the truncated NΔ294-ROS1 protein on these substrates. As expected, the N-terminal truncated protein was easier to dissociate from both SL1 and SL2 than the full-length version, but once again it did not show any differential behaviour in relation to the position of the obstacle (Figure 3, lower panel). Therefore, we conclude that ROS1 randomly slides along DNA in a process facilitated by its N-terminal domain.

### ROS1 sliding along DNA increases 5-meC excision

**Figure 4.**
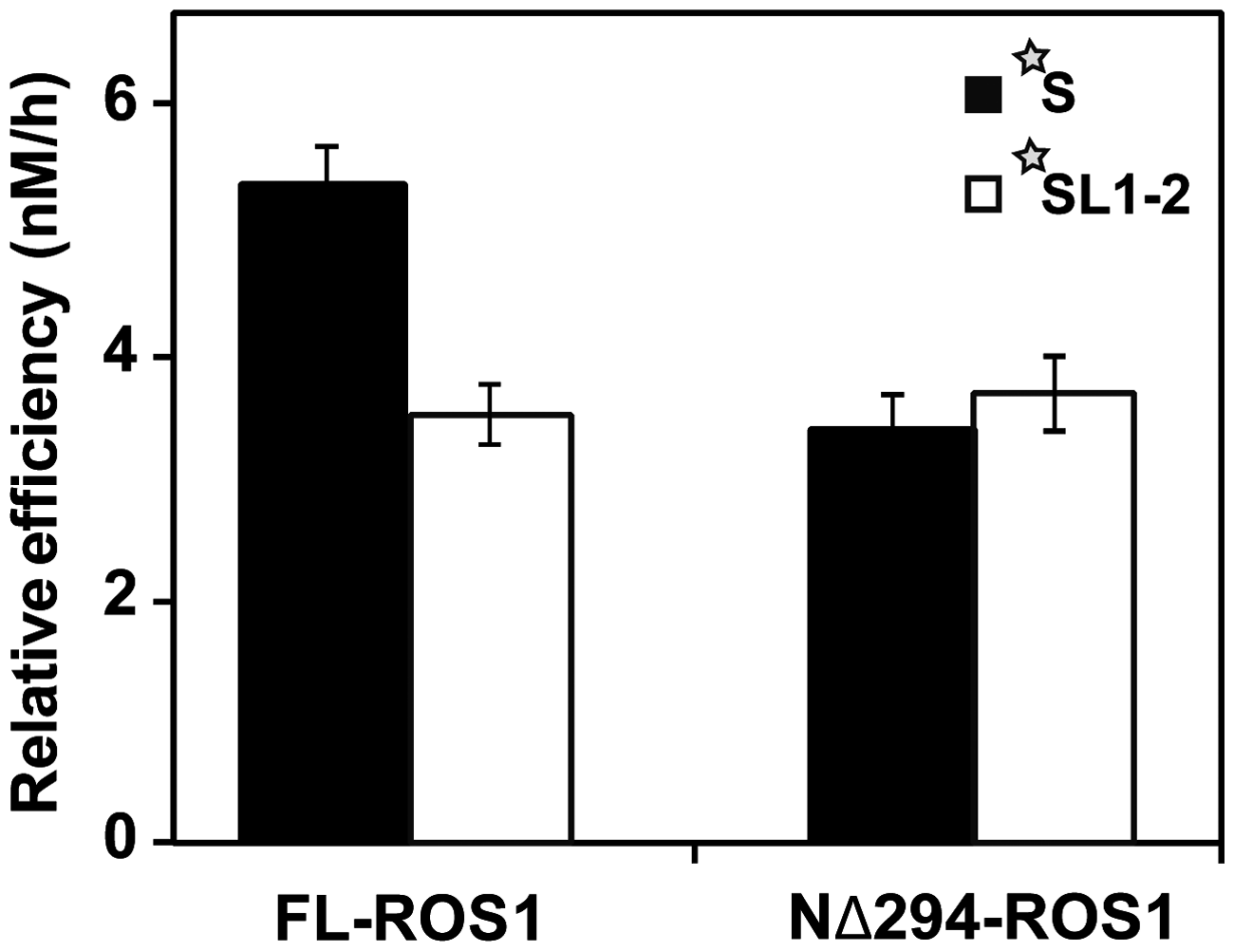
DNA sliding increases 5-meC excision efficiency. Purified proteins (20 nM) were incubated at 30°C with fluorescein-labelled substrates S and SL1-2 (20 nM) containing a single 5-meC:G pair. Reaction products were separated in a 12% denaturing polyacrylamide gel and quantified by fluorescence scanning. The relative processing efficiencies (E_rel_) were determined in kinetic assays as described in the experimental procedures (see Supplementary Table S2). Values are mean ± SE from two independent experiments, adjusted with the unbiased estimator described in (36).

We next asked whether the capacity of ROS1 to diffuse linearly on DNA facilitates 5-meC excision. We examined the ability of FL-ROS1 and its truncated version NΔ294-ROS1 to process substrates S and SL1-2, both containing a 5-meC:G at a central position in a 5′-CG-3′ context (Figure 4). We found that the relative processing efficiency of FL-ROS1 on DNA substrate SL1-2 was significantly lower than on substrate S (Figure 4 and Supplementary Table S2). In contrast, the relative efficiency of NΔ294-ROS1, although lower than that of the full-length protein, was similar on both substrates (Figure 4 and Supplementary Table S2).

These results agree with the idea that obstacles along DNA hinder access to 5-meC through sliding, forcing target detection by FL-ROS1 to approach a 3D search and reducing the catalytic efficiency of the enzyme. In the N-terminal truncated protein, stable binding to DNA and sliding capacity are reduced compared with the full-length version (as seen in the right panels of Figure 2B), therefore target location mainly relies on 3D encounters that are not affected by the presence of obstacles along the macromolecule. As a result, in substrate S, FL-ROS1 exhibits higher activity than NΔ294-ROS1 because the former is able to perform 1D search in addition to 3D encounters. However, in the substrate SL1-2, both proteins exhibit similar activity because in this substrate, sliding by FL-ROS1 is also severely restricted. Thus, limiting sliding, either by placing obstacles along DNA or by removing the N-terminus, leads to a similar reduction in catalytic activity. Altogether, these results support the hypothesis that the capacity to slide along DNA facilitates 5-meC excision by ROS1.

### Sliding does not facilitate ROS1 processing of T:G mispairs

ROS1 is a 5-meC DNA glycosylase but also excises T (=5-meU) from T:G mispairs, albeit with reduced efficiency (11,31). We have previously reported that non-specific DNA binding afforded by the N-terminus increases excision of 5-meC, but does not affect processing of its deamination derivative T (21). As the results described earlier indicate that the N-terminus favours a 1D diffusion process that in turn facilitates 5-meC excision, we decided to study the role of sliding on T excision.

**Figure 5.**
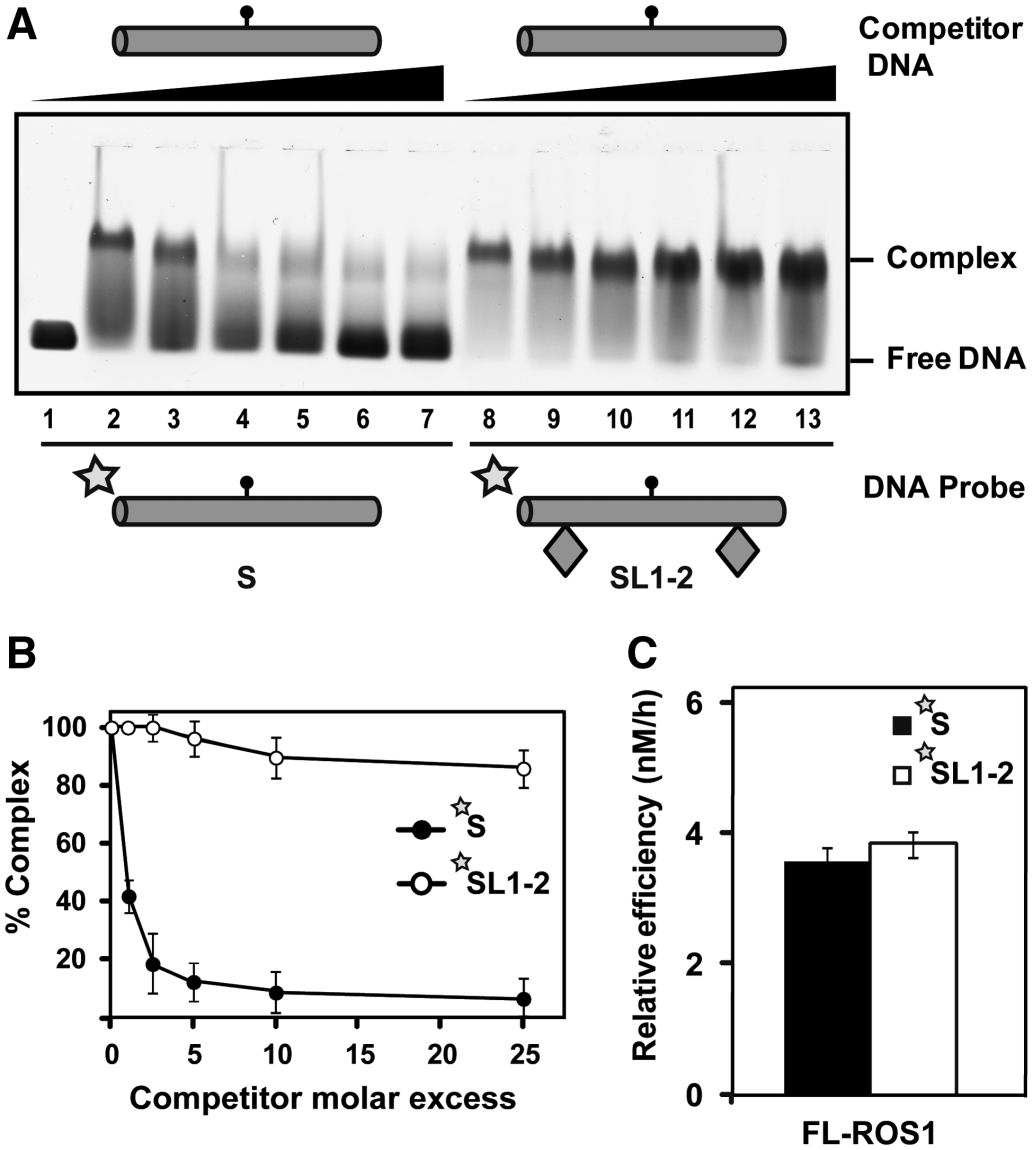
ROS1 linear diffusion along DNA does not facilitate processing of T:G mispairs. (**A**) Gel shift assay showing dissociation of FL-ROS1 (120 nM) from fluorescein-labelled substrates S (lanes 2–7, 100 nM) and SL1-2 (lanes 8–13, 100 nM) containing a T:G mispair on addition of increasing amounts (0, 0.1, 0.25, 0.5, 1.0 and 2.5 μM) of a mismatched unlabelled S competitor. After non-denaturing gel electrophoresis, the gel was scanned to detect fluorescein-labelled DNA. Protein-DNA complexes were identified by their retarded mobility compared with that of free DNA, as indicated. (**B**) Graph shows the percentage of remaining complex FL-ROS1-DNA versus competitor molar excess ratios. Values are mean ± SE from two independent experiments. (**C**) FL-ROS1 (20 nM) was incubated at 30°C with fluorescein-labelled substrates S and SL1-2 (20 nM) containing a single T:G mispair. Reaction products were separated in a 12% denaturing polyacrylamide gel and quantified by fluorescence scanning. Values are mean ± SE of the relative processing efficiencies (E_rel_) from two independent experiments, adjusted with the unbiased estimator described in (36).

We first investigated whether the presence of a T:G mispair exerts any effect on ROS1 capacity to slide along DNA. We performed an EMSA competition assay using substrates S and SL1-2 containing a central T:G mispair instead of a 5-meC:G pair (Figure 5A and B). We found that the behaviour of ROS1 on the mismatched substrates was similar to that observed on methylated DNA (Figure 2B, upper panel). Although the protein easily dissociates from the substrate with no obstacles, it remains bound to the substrate with obstacles even at high competitor concentrations (Figure 5A and B). We also confirmed that the inability of ROS1 to dissociate from a mismatched SL1-2 probe is not due to a higher affinity for this substrate (Supplementary Figure S8). These results strongly suggest that ROS1 also performs linear diffusion on DNA substrates containing T:G mismatches.

We next asked whether, similarly to 5-meC excision, the presence of obstacles at both sides of the target base negatively affects T excision. Interestingly, we found that the relative efficiencies of ROS1 on substrates S and SL1-2 containing a T:G mispair were similar (Figure 5C). This result suggests that, in contrast to 5-meC excision, hampering access to the mismatched T through sliding does not reduce the enzyme capacity to excise it. We therefore conclude that ROS1 achieves recognition and excision of its two target bases by different means. A model for location of potential target bases by ROS1 is proposed later in the text.

## DISCUSSION

Location of target 5-meC residues in the genome is likely to be the rate-limiting step during active DNA demethylation in plants, as ROS1 and its homologs face the difficult task to find a modified, but otherwise non-damaged, correctly paired base. Our previous work revealed that ROS1 binds unmethylated and methylated DNA with similar affinity (21), thus suggesting that facilitated diffusion along DNA may importantly contribute to target location by ROS1. In this work, we have critically tested this hypothesis by using DNA substrates containing obstacles on the DNA surface. We have found that ROS1 shows end-dependent dissociation that is severely restricted when access to the DNA ends is obstructed by tetraloops blocks. Although our results also show that dissociation may also occur by a 3D mechanism, they strongly suggest that 1D sliding is a major component of target location by ROS1. Our studies were performed with 61-bp substrates and therefore only provide information about short-range distances, but they indicate that ROS1 makes limited use of hopping or long-range jumping, at least *in vitro*.

By using DNA substrates blocked at either one or other end of the molecule, we have also found that target search by ROS1 is a directionally unbiased process. We speculate that such random sliding might help ROS1 to exhaustively interrogate the duplex by repeated visits to the same site within a DNA segment, increasing the probability to form a catalytic complex with its target base. The fact that ROS1 performs sliding on DNA to find its target base does not imply that the protein behaves as a processive enzyme. Processivity is a property arising from correlated cleavage, i.e. the probability of the enzyme resuming the walk after catalysis (a catalytic cycle) is complete (37), and therefore is strongly dependent on the rate of product release. Although ROS1 slides DNA in search for its target base, it actually excises 5-meC in a distributive fashion (31), because strongly binds to the single nucleotide gap generated after base excision (21).

In this study, we have also explored the molecular features of ROS1 responsible for sliding. Our previous results had already suggested that the basic, Lys-rich N-terminal region of ROS1 mediates non-specific binding to DNA and facilitates 5-meC excision on long molecules (21). In the present study, we found that the amino-terminal truncated protein is impaired in linear diffusion and easily dissociates from DNA molecules with blocked ends. These results suggest that the electrostatic interactions afforded by the basic N-terminal domain serve to hold the enzyme on the DNA and facilitate sliding. Our findings add to the growing evidence of an important role of low complexity, positively charged domains, in modulating linear diffusion of proteins along DNA (38). For example, the positively charged C-terminus of p53 promotes sliding along DNA (34), and the basic charge of the processivity factor UL42 endows the protein with the capacity to slide DNA in a tight, non-specifically bound state (35). The fact that ROS1 needs free DNA ends to dissociate from the macromolecule suggests that the enzyme engages in a genuine sliding mode that involves a persistent protein–DNA contact. The compromise between the strong binding afforded by the positively charged N-terminal domain and the ability to move along DNA may require a balance with repulsive forces on other regions of the protein, as previously suggested for other proteins performing linear diffusion (24).

Single-molecule methods allow for the direct observation of diffusion along DNA, but they have been criticized for using DNA substrates lacking the target site for the protein in question and/or for the need to stretch DNA out of its native configuration (39). In this study, we have used identical substrates both to test ROS1 diffusion along DNA and to analyse its catalytic activity. We have found that obstacles impede access to DNA ends and restrict ROS1 dissociation from DNA, but at the same time decrease the catalytic efficiency of 5-meC excision. The explanation is that they hinder the motion of the protein in both directions, both towards and away from the target site. Thus, they restrict ROS1 dissociation from DNA, but also prevent target access from initial binding sites placed away from the 5-meC:G central pair. The consequence of this latter effect is to effectively limit the size of the DNA substrate, thus approaching target location to a 3D search in solution. As a net result, obstacles along the DNA surface impede ROS1 sliding and decrease excision efficiency of 5-meC.

Our experimental approach allows testing the effect of sliding on different target residues. Interestingly, we have found that obstructing the sliding capacity of ROS1 decreases the catalytic efficiency on 5-meC:G pairs, but does not significantly affects processing of T:G mispairs. This result is in agreement with our previous enigmatic observation that removing the N-terminal domain notably reduces ROS1 activity on 5-meC, but not on T residues (21). Our results indicate that impairing the sliding capacity of ROS1 (either by placing obstacles along DNA or by removing the N-terminal domain) has dissimilar consequences for activity on 5-meC:G pairs and T:G mispairs, which suggests that ROS1 achieves recognition and excision of its two target bases by different mechanisms.

**Figure 6.**
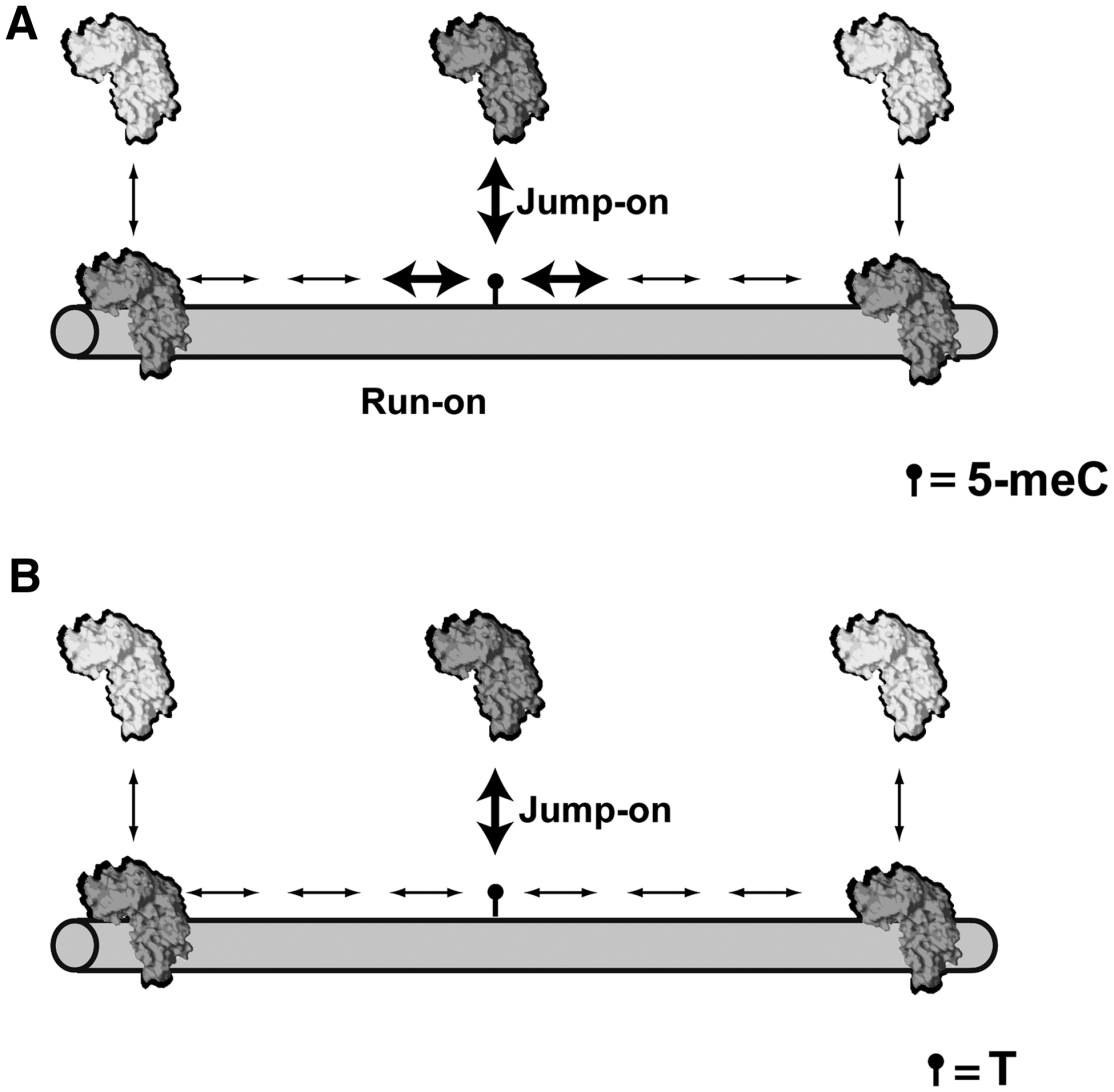
A model for 5-meC and T recognition by ROS1. The model is based on that proposed in (40) for DNA glycosylase Fpg, and postulates two recognition modes. In the ‘run-on mode’, the enzyme binds non-specifically and approaches its target by sliding along the DNA. In the ‘jump-on’ mode, the protein binds in the immediate vicinity of the target. ROS1 would form productive catalytic complexes in both modes with 5-meC:G pairs (**A**), but only in the ‘jump-on’ mode with T:G mispairs (**B**). Productive encounters are indicated by bold arrows, and protein motion is represented by thin arrows. See text for details.

A plausible model suggesting two different modes of lesion finding has been previously proposed for the DNA glycosylase Fpg, which excises 8-oxo-7,8-dihydroguanine and less efficiently 5,6-dihydrouracil (40). We have adapted this model to explain 5-meC and T recognition by ROS1 (Figure 6). The model postulates two modes of target location. In the ‘run-on’ mode, the enzyme approaches the target while sliding along DNA, whereas in the ‘jump-on’ mode, it binds in the immediate vicinity of the target. We propose that ROS1 forms productive catalytic complexes in both modes with 5-meC:G pairs, but only in the latter mode with T:G mispairs (Figure 6). Similarly to other DNA glycosylases, it is likely that during sliding, ROS1 forms a transient ‘interrogation complex’ that would extrude bases for inspection (41). We propose that such ‘interrogation complex’ would convert into a catalytically productive ‘excision complex’ on encountering 5-meC, but not T. This mechanism might limit the risk that an extruded correctly paired T attains the pre-catalytic state of the base extrusion pathway. Thus, the structural and mechanistic features of ROS1 might have evolved to simultaneously optimize excision of 5-meC and prevent aberrant activity on an overwhelmingly abundant suboptimal target.

## SUPPLEMENTARY DATA

Supplementary Data are available at NAR Online: Supplementary Tables 1 and 2 and Supplementary Figures 1–8.

## FUNDING

Spanish Ministry of Economy and Competitiveness; European Regional Development Fund [BFU2010-18838]; Junta de Andalucía, Spain [P07-CVI-02770], PhD Fellowship (to M.I.P.-M.). Funding for open access charge: Spanish Ministry of Economy and Competitiveness; European Regional Development Fund [BFU2010-18838].

*Conflict of interest statement*. None declared.

## Supplementary Material

Supplementary Data
