# Supplementary material for: Demethylation initiated by ROS1 glycosylase involves random sliding along DNA
Source: Nucleic Acids Res. 2012 Oct 2;40(22):11554–62. doi: 10.1093/nar/gks894 (PMC3526269; doi:10.1093/nar/gks894)
Supplement: Supplementary Data [file supp_40_22_11554__index.html]

Demethylation initiated by ROS1 glycosylase involves random sliding along DNA — Supplementary Data 

# Demethylation initiated by ROS1 glycosylase involves random sliding along DNA

## Supplementary Data

files

**Files in this Data Supplement:**

- Supplementary Data - pdf file
