# Supplementary material for: Demethylation initiated by ROS1 glycosylase involves random sliding along DNA
Source: Nucleic Acids Res. 2012 Oct 2;40(22):11554–62. doi: 10.1093/nar/gks894 (PMC3526269; doi:10.1093/nar/gks894)
Supplement: Supplementary Data [file supp_gks894_nar-01695-f-2012-File008.pdf]

Supplemental material for:

Demethylation initiated by ROS1 glycosylase involves random sliding along DNA

María Isabel Ponferrada Marín, Teresa Roldán-Arjona and Rafael R. Ariza

Department of Genetics, University of Córdoba/IMIBIC, Spain

Table S1
Table S2
Figure S1
Figure S2
Figure S3
Figure S4
Figure S5
Figure S6
Figure S7
Figure S8

Table S1. Oligonucleotides used as substrates

Name	DNA Sequence[a]	Strand	X=	Length
FL-meCGF61	5´-TCACGCGGGATCAATGTGTTCTTTCAGCTC**X**GGTCACGCTGACCAGGAATACCTCACTACC-3´	Upper	5-me	61
FL-CGF61	5´-TCACGCGGGATCAATGTGTTCTTTCAGCTC**X**GGTCACGCTGACCAGGAATACCTCACTACC-3´	Upper	C	61
FL-TGF61	5´-TCACGCGGGATCAATGTGTTCTTTCAGCTC**X**GGTCACGCTGACCAGGAATACCTCACTACC-3´	Upper	T	61
CGR61	3´-AGTGCGCCCTAGTTACACAAGAAAGTCGAGG**X**CAGTGCGACTGGTCCTTATGGAGTGATGG-5´	Lower	C	61
CGR61 LOOP1	3´-AGTGCGCCCTAGTTACACAAGAAAGTCGAGG**X**CAGTGCGACTGGTaggaatgcctgggaatgccccCCTTATGGAGTGATGG-5´	Lower	C	81
CGR61 LOOP2	3´-AGTGCGCCCTAGTTACaggaatgcctgggaatgcccACAAGAAAGTCGAGG**X**CAGTGCGACTGGTCCTTATGGAGTGATGG-5´	Lower	C	81
CGR61 LOOP1-2	3´-AGTGCGCCCTAGTTACaggaatgcctgggaatgcccACAAGAAAGTCGAGG**X**CAGTGCGACTGGTaggaatgcctgggaatgccccCCTTATGGAGTGATGG-5´	Lower	C	101

[a] Relevant regions are boxed. Lower case indicates the presence of an obstacle created in the DNA substrate.

Table S2. Relative substrate processing efficiencies FL-ROS1 and NΔ294–ROS1 on substrates containing a single 5-meC:G pair

	S			SL1-2		
	P_{max} (nM)	$T_{1/2}$ (h)	E_{rel} [a]	P_{max} (nM)	$T_{1/2}$ (h)	E_{rel} [a]
FL-ROS1	13,20 ± 0,63	2,33	5,66 ± 0,27	11,10 ± 0,62	3,03	3,67 ± 0,20
NΔ294–ROS1	7,46 ± 0,51	2,09	3,57 ± 0,24	4,14 ± 0,28	1,06	3,91 ± 0,27

[a] $P_{max}/T_{1/2}$

FIGURE S1

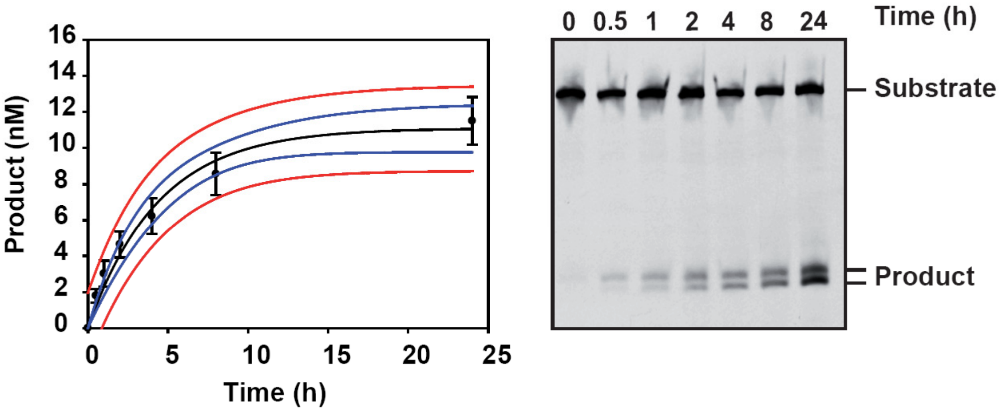

Figure S1. Representative example of 5-meC DNA glycosylase assay and kinetic analysis. The time-dependent generation of incision products was measured by incubating purified FL-ROS1 (20 nM) at 30 °C with a fluorescein-labeled substrate SL1-2 (20 nM) containing a single 5-meC:G pair. Reactions were stopped at the indicated times, products were separated in a 12% denaturing polyacrylamide gel and quantified by fluorescence scanning. The graph shows the generation of incision products versus time. Values are means ± S.E. (error bars) from two independent experiments. Data were fitted to the equation [Product] = $P_{max}[1\text{-}exp^{(-kt)}]$ using non-linear regression analysis. Blue and red curves indicate 95% confidence and prediction intervals, respectively.

FIGURE S2

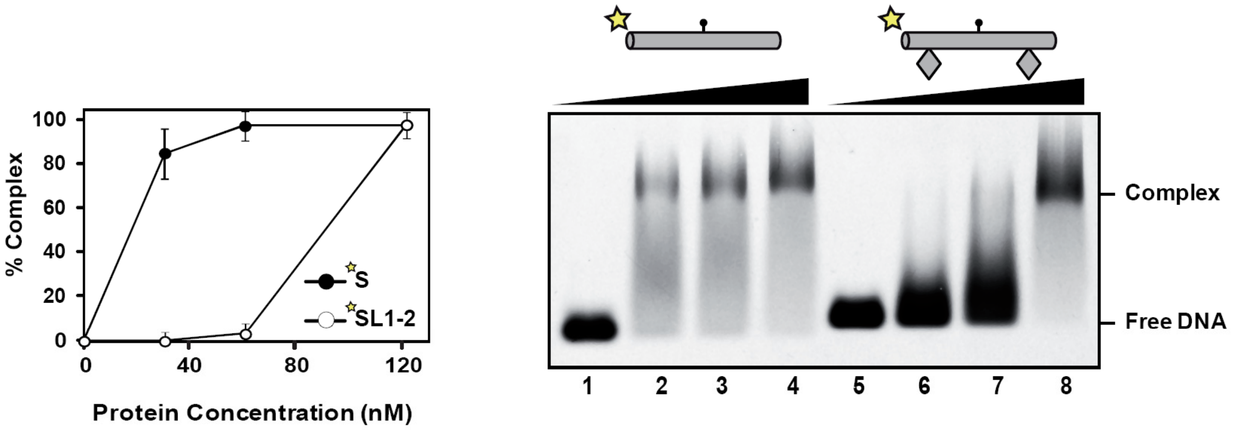

Figure S2 ROS1 binding to substrates S and SL1-2. Increasing concentrations of FL-ROS1 (0, 30, 60, and 120 nM) were incubated with fluorescein-labeled substrates S (lanes 1-4, 100 nM) and SL1-2 (lanes 5-8, 100 nM) containing a single 5-meC:G pair. After nondenaturing electrophoresis, the gel was scanned to detect fluorescein-labeled DNA. Protein-DNA complexes were identified by their retarded mobility compared with that of free DNA, as indicated. Graphs on the left show the percentage of complex versus protein concentration. Values are mean ± SE from two independent experiments, adjusted with the unbiased estimator described in (41).

FIGURE S3

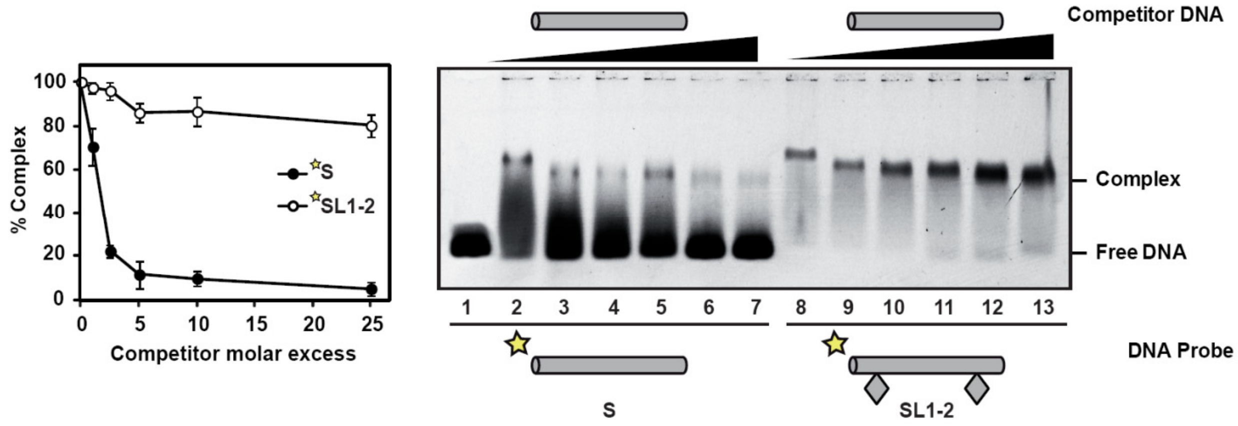

Figure S3. ROS1 performs sliding on homoduplex DNA. Gel shift assay showing dissociation of FL-ROS1 (120 nM) preincubated for 5 min with fluorescein-labeled homoduplex S (lanes 2-7, 100 nM) or SL1-2 (lanes 8-13, 100 nM) upon addition of increasing amounts (0, 0.1, 0.25, 0.5, 1.0, and 2.5 μM) of unlabeled homoduplex competitor S. After nondenaturing gel electrophoresis, the gel was scanned to detect fluorescein-labeled DNA. Protein-DNA complexes were identified by their retarded mobility compared with that of free DNA, as indicated. Graphs on the left show the percentage of remaining complex versus competitor molar excess ratios. Values are mean ± SE from two independent experiments, adjusted with the unbiased estimator described in (41).

FIGURE S4

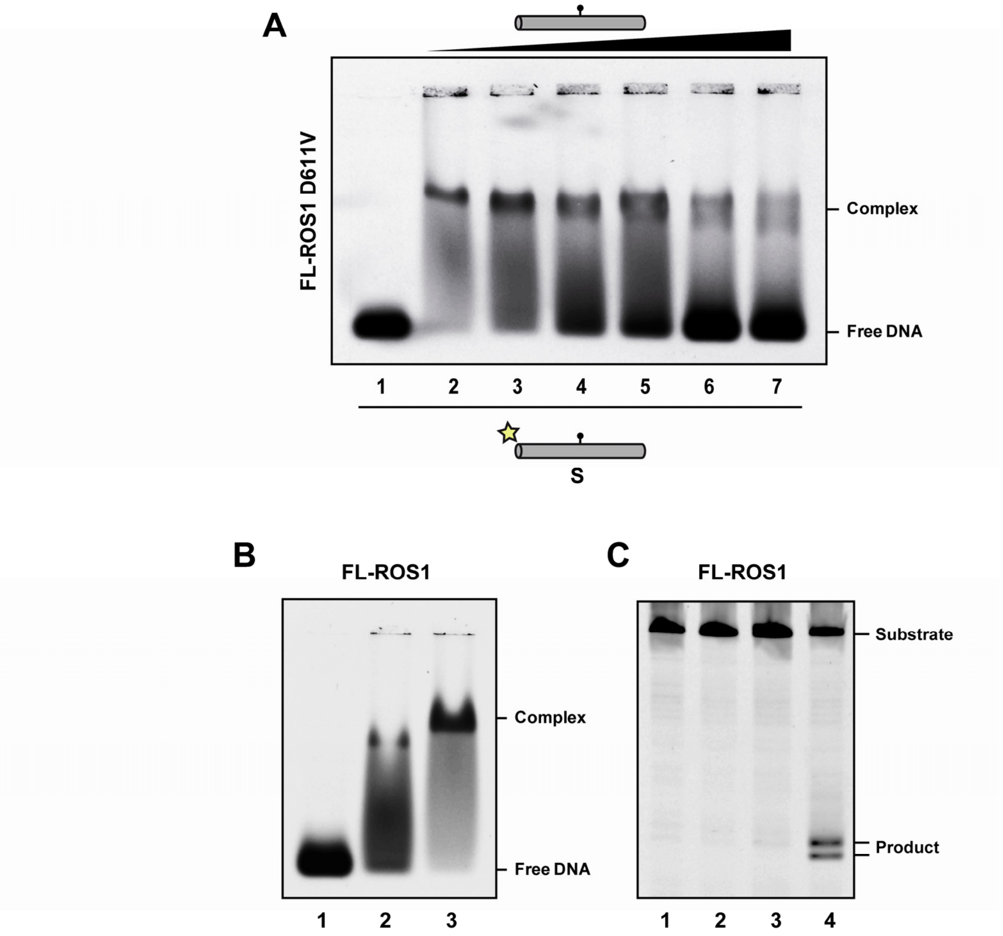

Figure S4. DNA sliding is independent of catalytic activity. A. Gel shift assay showing dissociation of mutant FL-ROS1 D611V (120 nM), which lacks catalytic activity (20), from fluorescein-labeled substrate S (lanes 2-7, 100 nM) containing a single 5-meC:G pair upon addition of increasing amounts (0, 0.1, 0.25, 0.5, 1.0, and 2.5 μM) of unlabeled methylated competitor S. After nondenaturing gel electrophoresis, the gel was scanned to detect fluorescein-labeled DNA. Protein-DNA complexes were identified by their retarded mobility compared with that of free DNA, as indicated. **B.** Gel shift assay with WT-FL-ROS1. Increasing concentrations of WT FL-ROS1 (0, 20, and 120 nM) were incubated at 25º C with fluorescein-labeled substrate S (lanes 1-3, 100 nM) containing a single 5-meC:G pair in EMSA conditions (see Materials and Methods). After nondenaturing electrophoresis, the gel was scanned to detect fluorescein-labeled DNA. Protein-DNA complexes were identified by their retarded mobility compared with that of free DNA, as indicated. **C.** DNA glycosylase activity was measured in EMSA reactions performed in parallel to those shown in B (lanes 1-3). A control DNA glycosylase reaction (lane 4) was performed by incubating FL-ROS1 (20 nM) at 30ºC for 1h with the same DNA substrate (20 nM) in standard DNA glycosylase assay conditions (see Materials and Methods).

FIGURE S5

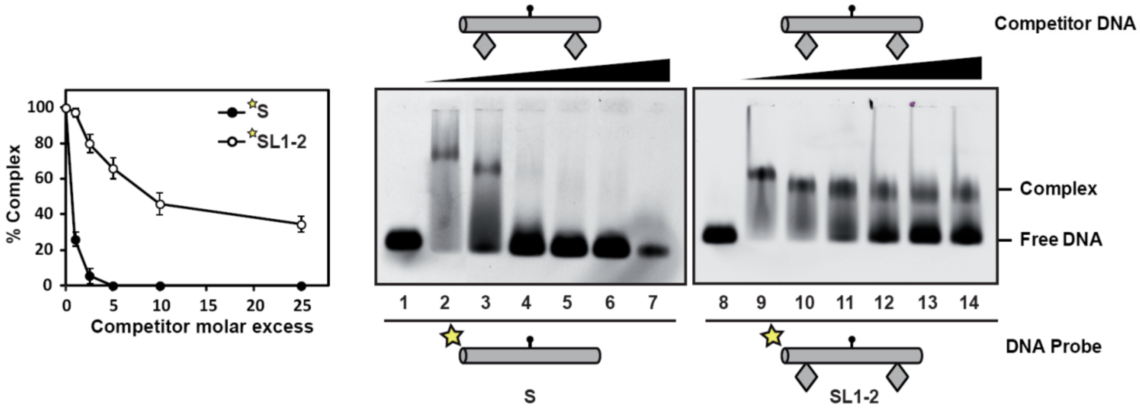

Figure S5. ROS1 dissociation from S and SL1-2 substrates in the presence of unlabeled competitor SL1-2. Gel shift assay showing dissociation of FL-ROS1 (120 nM) from fluorescein-labeled substrates S (lanes 2-7, 100 nM) and SL1-2 (lanes 9-14, 100 nM) containing a single 5-meC:G pair upon addition of increasing amounts (0, 0.1, 0.25, 0.5, 1.0, and 2.5 μM) of unlabeled methylated competitor SL1-2. After nondenaturing gel electrophoresis, the gel was scanned to detect fluorescein-labeled DNA. Protein-DNA complexes were identified by their retarded mobility compared with that of free DNA, as indicated. Graphs on the left show the percentage of remaining complex versus competitor molar excess ratios. Values are mean ± SE from two independent experiments, adjusted with the unbiased estimator described in (41).

FIGURE S6

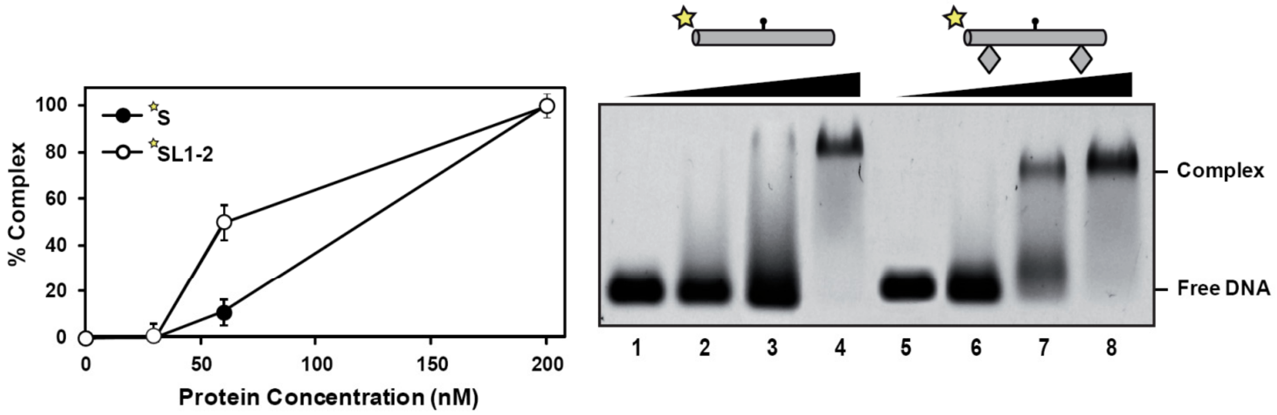

Figure S6. NΔ294-ROS1 binding to substrates S and SL1-2. Increasing concentrations of NΔ294-ROS1 (0, 30, 60, and 200 nM) were incubated with fluorescein-labeled substrates S (lanes 1-4, 100 nM) and SL1-2 (lanes 5-8, 100 nM) containing a single 5-meC:G pair. After nondenaturing electrophoresis, the gel was scanned to detect fluorescein-labeled DNA. Protein-DNA complexes were identified by their retarded mobility compared with that of free DNA, as indicated. Graphs on the left show the percentage of complex versus protein concentration. Values are mean ± SE from two independent experiments, adjusted with the unbiased estimator described in (41).

FIGURE S7

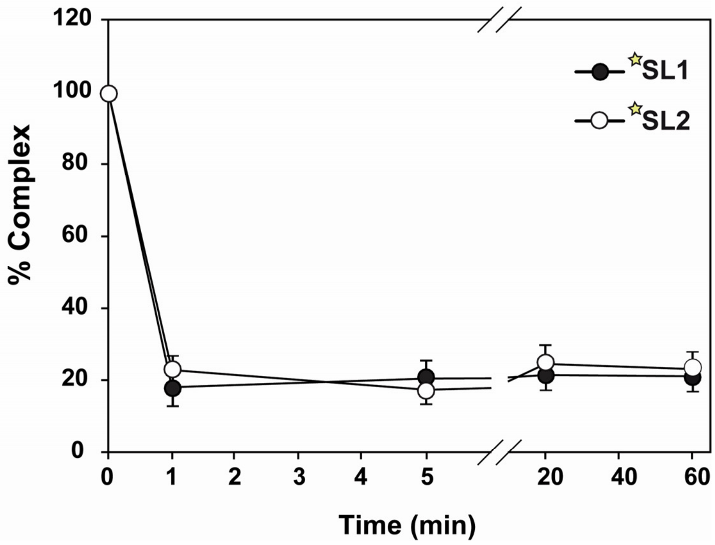

Figure S7. Dissociation kinetics of ROS1 from SL1 or SL2 DNA substrates. Graph shows the percentage of remaining protein-DNA complex of FL-ROS1 (120 nM) preincubated for 5 min with fluorescein-labeled methylated substrates SL1 and SL2, at different times upon addition of 0.5 μM unlabeled methylated competitor SL1-2. Values are mean ± SE from two independent experiments, adjusted with the unbiased estimator described in (41).

FIGURE S8

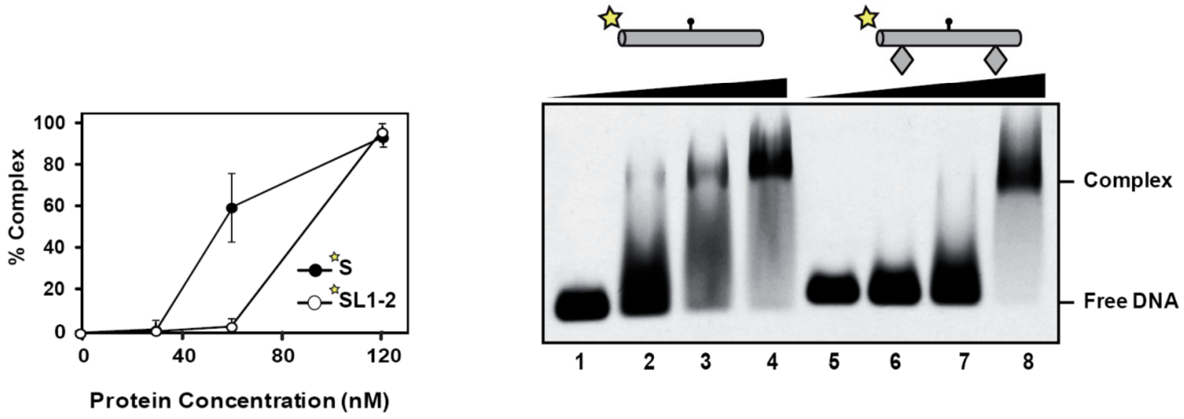

Figure S8. ROS1 binding to substrates S and SL1-2 containing a T:G mismatch. Increasing concentrations of FL-ROS1 (0, 30, 60, and 120 nM) were incubated with fluorescein-labeled substrates S (lanes 1-4, 100 nM) and SL1-2 (lanes 5-8, 100 nM) containing a single T:G mispair. After nondenaturing electrophoresis, the gel was scanned to detect fluorescein-labeled DNA. Protein-DNA complexes were identified by their retarded mobility compared with that of free DNA, as indicated. Graphs on the left show the percentage of complex versus protein concentration. Values are mean ± SE from two independent experiments, adjusted with the unbiased estimator described in (41).
